# AIDS and recurrent aphtous stomatitis

**DOI:** 10.1016/S1808-8694(15)31209-X

**Published:** 2015-10-20

**Authors:** Ivan Dieb Miziara, Bernardo Cunha Araujo Filho, Raimar Weber

**Affiliations:** 1 Professor (Ph.D) Department of Otorhinolaryngology, Medical School- USP, Technical Director of Health Care Service.; 2 Otorhinolaryngologist, internship at HCFMUSP, Specialized in Otorhinolaryngology , SBORL.; 3 Internist Physician.

**Keywords:** recurrent stomatitis, mouth disease, HIV infection, AIDS

## Abstract

The immunodeficiency state in HIV infected patients has been the cause of severe episodes of Recurrent Aphthous Stomatitis (RAS). **Aim**: Our study aims to establish correlation between the manifestations of RAS and the immunosuppression state caused by HIV infection, through counting of CD4+ cells, CD8+ cells, CD4+:CD8+ cells ratio and viral load. **Study design:** series study. **Material and Method**: Ninety-four HIV infected patients (25 women and 69 men) with RAS were evaluated in the ENT Department of the University of Sao Paulo-Medical School from January 1998 to December 2003. The age ranged between 19 and 63 years (mean = 35.3 years). The patients were divided in two groups: AIDS group and HIV infected group. **Results**: The patients with AIDS and HIV infection presented, respectively, eight ulcers and two ulcers by outbreaks. Similarly, patients with major RAS presented smaller counting of cells CD8+, CD4+ and CD4+/CD8+ cells, and higher mean value of viral load than the patients with herpetiform and minor RAS. Between patients with minor and herpetiform RAS there were no statistical differences. **Conclusions**: The emergence of the lesions, mainly in major RAS, is directly related to the immunological state of the HIV infected patient. These patients frequently present nutritional deficits and worsening in life style. Thus, diagnosis and treatment of RAS is a challenge that should not be neglected.

## INTRODUCTION

Recurrent Aphthous Stomatitis (RAS) is a disorder characterized by painful ulcers with variable size and duration typically found in nonkeratinized sites of the oral mucosa. The three major types of aphtous stomatitis are minor, major and herpetiform. They are differentiated by number, size and duration, however such differentiation is not always clear^1^, ^2^.

In general, minor type aphtous stomatitis measures less than 10mm and lasts on average from 10 to 14 days and is more common than the others. The major type Aphthous stomatitis (ulcers size ranging from 10-30mm) is less common and more severe, lasts longer (weeks and months), and usually results in scars.

The herpetiform types of ulcers (1 to 3mm size) are less frequent^2^, ^3^ and the number of ulcers can be highly increased up to 100 ulcers per episode. Its name derives from its alleged similarity with oral ulcers caused by primary herpetic gingivostomatitis, but Herpes Simplex Virus (HSV) could not be isolated in the ulcers.

Severe episodes of RAS have been reported in patients with human immunodeficiency virus (HIV) 2, 4, 5. Typically the ulcers have three types and are commonly located in the soft palate, tonsillar sites or tongue. Although many of such patients referred RAS in childhood, its frequency decreased with age and became more severe after symptomatic HIV infection. Most of the infected patients did not remember having any episode of RAS before acquiring HIV. Abnormalities in lymphocyte population of such patients have been considered as the etiological hypothesis for such lesions^6^, ^7^, but there are no available studies relating the disease to patient’s viral load.

The objective of this study was to establish evidences between the onset (or increase in severity) of RAS related to HIV immunosuppresion through CD4^+^, CD8^+^ cell count and viral load, and to relate the type of lesion found to immune status of the patient.

## MATERIAL AND METHODS

A consecutive group of 94 positive HIV patients (1) (25 female and 69 male), with major, minor and herpetiform RAS were followed up in the AIDS Outpatient Unit of the Clinical ENT Department of Hospital das Clínicas - FMUSP from January 1998 through December 2003. Patients’ ages ranged from 19 to 63 years (mean age= 35.3 years).

Direct Immunofluorescence tests were performed in skin swab using direct Syva MicroTrak HSV-1/HSV-2 (Syva Co., Palo Alto, Calif.) to rule out the etiological causes of ulcers related to herpes virus (HSV) and neutropenia as recommended by MacPhail et al. (1991), followed by complete blood test.

Blood samples were collected for CD4^+^, CD8^+^ cells count and viral load in the first appointment and subsequent follow up visit.

Twenty patients were excluded from the study (8 female and 12 male) since they had neutrophil and blood count below the normal values (1800 to 6800/mm3)/, and other 12 patients (3 female and 9 male) that tested positive for HSV in direct identification test.

All the remaining patients (62 patients) tested positive for HIV were evaluated at least twice a week within an interval of 30 days between each appointment. During the first appointment patients answered a questionnaire in order to record a thorough history of oral and past systemic diseases, including drugs currently in use. The questionnaire also included presence or absence of RAS in childhood and teenage years and if the answer was affirmative, patients should inform its characteristics related to number, frequency, duration, size, location and painful symptoms associated with it.

Patients underwent a thorough ENT examination. The presence of any oral disease was recorded, including the number, size, location and duration of current apthae.

The ulcers were classified as minor, major or herpetiform based on its size and amount per episode.

Biopsy was performed in oral ulcers with a punch higher than 6mm. The collected sample was formalin-fixed (10% formalin) and stained in hematoxillin and eosin and examined under optical microscopy.

First, the patients were divided into two groups. The first group with 35 patients met the criteria of the Center for Disease Control in Atlanta (CDC - 93) for acquired immunodeficiency syndrome (AIDS). The second group with 27 patients was formed by HIV positive cases.

In each one of the two groups (Aids and HIV-positive) the predominant type of ulcer was recorded, including the number of ulcers per episode. Results were expressed in percentage and submitted to statistical analysis applying Student’s T-test.

Subsequently, patients were divided in three new groups according to the type of ulcer they had (major, minor or herpetiform). Mean values of CD4^+^, CD8^+^ cell counts and viral load in each sub-group were statistically compared applying the Student’s T-test.

## RESULTS

The number of patients per type of aphtous ulcer, as well as the mean CD4+, CD8+ cell count and mean CD4+/CD8+: viral load ratio are listed in Table 1.

**Table 1:** Correlation between types of apthae, number of patients, lymphocyte and viral load count in HIV patients.

Type of apthae	Total number of patients	Number of patients with AIDS	Number of positive HIV patients	CD4 (mean)	CD8 (mean)	CD4/CD8 (mean)	Viral Load (mean)
Major	18	6	12	123	610	0,29	163420
				(15-367)	(32-4204)		(66322-356731)
Minor	25	10	15	346	948	0,3	18744
				(41-932)	(208-2324)		(2610-82244)
Herpetiform	10	3	7	295	826	0,4	23869
				(22-645)	(86-1867)		(4260-136254)
Mixed	9	2	7	327	812	0,35	20456
				(12-544)	(301-2103)		(3008-124749)

All types of ulcers were found in this study as follows: 29.03% of patients with isolated major type ulcers, 40.32% with minor type ulcer, and 16.2% with herpetiform type. The remaining patients (9/62) had different types of apthae in different appointments or in the same appointment.

All pathophysiological analysis detected only the presence of ulcerative lesion in oral mucosa followed by inflammatory infiltrate in the sub mucosa.

Patients with major and herpetiform type ulcers had never had the lesion before. – 54.83% of patients (34/62) did not remember having RAS in childhood. The remaining patients (28/62, 45.16%) reported RAS in childhood characterizing apthae as minor.

Patients with Acquired Immunodeficiency Syndrome (Aids) (CDC-93) had three types of oral ulcers. In each group, it accounted for less than half of the patients. There was not a most common type of aphtous ulcer found between patients with Aids and HIV-positive.

However, patients with Aids had an increased number of apthae per episode against those patients only HIV-positive disease. The mean number of apthae per episode in patients with Aids was eight (8), whereas in HIV-positive patients it was two (2). The difference was statistically significant (p=0.002).

Lymphocyte T CD4^+^ count showed a statistically significant relation with the RAS type. Patients with major type apthae compared against patients with minor type lesions had lower CD4^+^ cell count than those patients with herpetiform apthae (p=0.002) and patients with minor apthae (p=0.001).

On the other hand, the comparison between patients with herpetiform and minor ulcers did not show statistically significant difference in CD4^+^ cell count (p=0.053).

Likewise, patients with major apthae presented lower CD8^+^ cell count if compared against patients with herpetiform apthae (p=0.002) and minor apthae (p=0.001). There was no statistically significant difference between patients with herpetiform and minor apthae (p=0.006).

As regards to CD4^+^/CD8^+^ ratio, patients with major apthae had a lower mean against those patients with minor and herpetiform apthae. This difference, however, was not statistically significant. There was no statistically significant difference related to CD4^+^ or CD4^+^/CD8^+^ ratio in patients with herpetiform and minor apthae.

With respect to absolute CD4^+^ cell count, 55.5% (10/18) of the patients with major type apthae had less than 60 cells/mm^3^, against only 20% of the patients with herpetiform and minor apthae (7/35) that had cell count below 60 cells/mm^3^. This difference was statistically significant (p=0.002).

The onset of major ulcers, if compared with the onset of minor or herpetiform ulcers, was also associated with a higher mean value of viral load. This difference was statistically significant (p=0.002).

The comparison of patients with minor and herpetiform apthae against the mean viral load was not statistically different (p=0.056).

## DISCUSSION

Three types of apthae were found in the studied population. Total number of patients that presented major and herpetiform type were 45.5%. This finding was slightly different from that of MacPhail et al. ^2^, which reported 66% of the HIV patients with major and herpetiform aphtous ulcers.

The results of this study were also different from those of other research studies carried out related to the same topic in non-HIV patients, which estimated incidence level of 80% of minor type apthae and 20% of major and herpetiform type apthae^8^, ^9^.

However, there is an increased likelihood that the cause of such incidence of major and herpetiform type aphtous ulcer could be related to HIV infection itself and the subsequent immunosuppression of our patients, since none of them remembered having ulcers of such type during childhood.

The association of major apthae with immunosuppression degree becomes clearer whenever those patients with this type of aphtous ulcer have lower CD4^+^ and CD8^+^ cell count and increased inversion in the CD4^+^/CD8^+^ ratio against those patients with minor and herpetiform aphtous ulcer.

Interestingly enough, the number of patients with major apthae and total CD4^+^ cell count below de 60 was higher in those patients with herpetiform and minor apthae.

Some authors believe that RAS represents a local defect in immunomodulation with abnormalities in lymphocyte population (both blood stream and aphtous ulcer lymphocytes) resulting in a T CD8^+^ cell “attack” and subsequent destruction of the epithelium 10, 11, 12.

Since HIV infection is characterized by a progressive decline of CD4^+^ cell count and inverted CD4^+^/CD8^+^ ratio such as “relative increase” of CD8^+^ cells (mainly in ulcerative stage) it would result in a local defect amplification in the immunoregulation^13^.

Another interesting aspect is that although no prevalence of any type of apthae was found between any patient with AIDS and HIV-positive, the number of lesions per episode was higher in patients with Aids than in those patients with HIV positive, which may be an indicator of the correlation between immunosuppressant and increased severity of RAS.

Finally, it is important to point out that in this study the patients with major type apthae presented an increased mean of viral load against those patients with herpetiform and minor apthae. The authors do not know so far any other study in the literature that related viral load to the type of apthae found in patients. In our opinion viral load in patients with major aphtous ulcers could be directly related to accelerated decrease of CD4^+^ cells and inversion of the normal CD4^+^/CD8^+^ ration resulting in the onset of more severe lesions.

All patients in this study were treated with thalidomide (200mg/day) or dapsone (100mg/day) plus topical steroid drug (triancinolone in orabase ointment). In more severe cases the drug chosen was prednisone (60mg/day) per week until complete regression of lesions^13^.

## CONCLUSION

RAS-related morbidity is significant. The onset of lesions, primarily the major apthae, is directly related to the immune status of the HIV patient. These lesions result in inadequate eating and speaking for the patients, leading to nutritional deficits and worsening the quality of life. Since new anti-retroviral drugs have been prolonging survival rate, the patients with RAS diagnosis should not be neglected and treatment still remains a challenge even in relative states of immunosuppresion.
